# Evaluating prognostic value of biliary stone in intrahepatic cholangiocarcinoma by propensity score matching analysis

**DOI:** 10.7150/jca.74275

**Published:** 2023-05-08

**Authors:** Yupeng Wang, Ao Huang, Dezhen Guo, Jian Wang, Feiyu Chen, Huichuan Sun, Shuangjian Qiu, Sunyue Zhang, Ya Cao, Xinrong Yang, Jian Zhou

**Affiliations:** 1 Department of Liver Surgery and Transplantation, Liver Cancer Institute, Zhongshan Hospital, Fudan University, Shanghai, 200437, China.; 2 Key Laboratory of Carcinogenesis and Cancer Invasion, Fudan University, Shanghai, 200437, China.; 3 Cancer Research Institute, Central South University; Key Laboratory of Carcinogenesis and Cancer Invasion, Ministry of Education, Changsha, 410078, China.

**Keywords:** Intrahepatic Cholangiocarcinoma, Hepatolithiasis, Prognosis, Preoperative peripheral inflammation index, Tumor infiltrating immunocytes

## Abstract

**Background:** To explore biliary tract stone (BTS) as prognostic factors of intrahepatic cholangiocarcinoma (ICC).

**Methods:** Clinical data of 985 ICC patients were classified into no BTS group and BTS group-subgrouped into hepatolithiasis (HL) and non-hepatolithiasis (NHL) group. Propensity score matching was utilized to mitigate baseline characteristics. Preoperative peripheral inflammation parameters (PPIP) were further investigated. Immunostaining of CD3, CD4, CD8, CD68, PD1 and PD-L1 were conducted.

**Results:** Overall survival (OS) of patients without BTS surpassed BTS group (*P* = 0.040) while no difference of time to recurrence (TTR) was observed (*P* = 0.146). HL group had shorter OS and TTR than HL-matched group (*P* < 0.001 and *P* = 0.017, respectively) and survival time of NHL group differed not with NHL-matched group (*P* > 0.05). PPIP like neutrophils to lymphocytes ratio (NLR), platelet to lymphocyte ratio (PLR) and systemic immune inflammation (SII) of HL group exceeded no BTS group or NHL group (all *P* < 0.05). Associations of PPIP and tumorous immunocytes differed vastly among HL group, NHL group and no BTS group. Tumorous CD4^+^/CD3^+^ ratio and PD1^+^/CD3^+^ ratio of HL group surpassed those in no BTS group (*P* = 0.036 and *P* < 0.001, respectively) and NHL group (*P* = 0.015 and 0.002, respectively). Para-tumorous CD68^+^ macrophages exceeded that in tumor samples of HL group (*P* < 0.001). No difference of CD8^+^/CD3^+^ lymphocyte ratio and PD-L1 rank were detected.

**Conclusions:** Hepatolithiasis, rather than extra-hepatic biliary stone, is a poor prognostic indicator of ICC. Immunotherapy is promising in treating HL-related ICC.

## Introduction

Intrahepatic cholangiocarcinoma (ICC) is the second most common primary liver cancer 1. As a highly lethal malignancy, though surgery remains the mainstay of curative treatment 2, the postoperative 5-year survival rate can be as low as 30% 3. Known epidemiological risk factors of ICC are hepatitis B/C virus infection, hepatolithiasis, liver fluke infection etc. 1. As etiologically different ICC patients vary in tumorigenesis, which probably lead to diverse postoperative prognoses, it's imperative to clarify the underlying mechanisms and thus aid to enable early detection and intervention.

Biliary tract stone (BTS) can be classified into intra-hepatic biliary stone (hepatolithiasis, HL) and extra-hepatic biliary stone according to the anatomical position. Formation of BTS is facilitated by bile stasis and both intra-hepatic and extra-hepatic bile tract stone share similar causes like obesity, hyperlipidemia, genetic background and bacterial infection of biliary tract 4, 5 and may result in unfavorable inflammatory microenvironment. Hepatolithiasis has been recognized as a risk factor of ICC 6, 7 as HL possibly induces persistent mechanical stress and chronic inflammation of biliary epithelium 5, however, the underlying mechanism still needs to be further clarified. Additionally, cholecystolithiasis is proposed to be a possible risk factor of ICC^6^ as well, it's of great clinical value to appraise the role of BTS in predicting prognoses of ICC patients.

Immune escape and inflammation are two hallmarks of cancer, and tumor cells paralyze components of immune systems such as CD8^+^ cytotoxic T cells which are dispatched to eradicate them 8. Therapies against PD1/PD-L1 exhibit great favorable responses in various solid tumors including cholangiocarcinoma 9-11. Moreover, preoperative peripheral inflammation parameters (PPIP) that reflect systemic inflammation and immune response like neutrophil to lymphocyte ratio (NLR), platelet to lymphocyte ratio (PLR), systemic immune inflammation (SII), lymphocyte to monocyte ratio (LMR) and systemic inflammation response index (SIRI) were reported to be prognostic predictors in various solid tumors including ICC 12-16. Interestingly, cholelithiasis often occur with cholestasis which causes inflammation of biliary epithelium 17 and neutrophils were confirmed to promote the formation and growth of gallstone, indicating activation of immune system is required during formation of BTS 18. However, there exists scarce report concerning changes in the tumor microenvironment of cholelithiasis-related ICC so far.

In this study, we retrospectively reviewed clinical characteristics and prognoses of a hospital-based ICC cohort with different subtypes of BTS, and then we analyzed PPIP among those groups. Furthermore, markers of immunocytes were stained and then compared to further the understanding of mechanism of poor prognoses of subgroups of cholelithiasis-related ICC.

## Materials and Methods

### Patients and design

From January 2005 to December 2015, 1129 patients treated with curative-intention hepatectomy and pathologically confirmed to be cholangiocarcinoma were reviewed at the Department of Liver Surgery and Transplantation, Liver Cancer Institute, Zhongshan Hospital, Fudan University. Then 985 patients meeting the inclusion criteria were enrolled in the present study. Written informed consents were signed by each patient and ethical approval was acquired from Zhongshan Hospital Research Ethics Committee in accordance with the Declaration of Helsinki (revised in 2013). Inclusion criteria: (1) complete removal of neoplasm with negative microscopic margin; (2) pathologically diagnosed as ICC based on World Health Organization criteria. Exclusion criteria: (1) trans-catheter arterial embolization or radio frequency ablation or other types of anti-cancer treatment before the surgery (n = 35); (2) diagnosed with any other neoplasms preoperatively (n = 28); (3) recurrent ICC patients (n = 47); (4) metastasis found intra-operatively (n = 34).

Totally, 985 subjects were divided into biliary stone negative group (n = 843) and biliary stone positive group (n = 142), and the latter was further classified into hepatolithiasis (HL) group (n = 51) including 29 patients with simple hepatolithiasis, 9 patients with both hepatolithiasis and gallbladder stone (GBS), 3 patients with both HL and common bile duct stone (CBS), 10 patients with both HL, GBS and CBS, and non-HL (NHL) group (n = 91) including 3 patients with both GBS and CBS, 85 patients with simple GBS, 3 patients with simple CBS. Moreover, the survival time of the whole cohort was also analyzed according to epidemiological factors: (1) HBV positive group (n = 601): HBV positive was judged as previously defined^2^ without BTS or fluke infection; (2) fluke positive group (n = 26): pathologically confirmed the existence of ova of fluke or medical records of fluke infection without HBV infection or BTS; (3) stone-related group (n = 35): ICC patients with imageologically or pathologically diagnosed with BTS, and no infection of HBV or liver fluke; (4) cryptogenic group (n = 185): ICC patients without the above risk factors; (5) dual/triple positive group (n = 138): any two or three factors positive simultaneously.

### Follow-ups

Survival data, including overall survival (OS) and time to recurrence (TTR), were collected until December, 2018. Data were censored at the last follow-up for surviving patients. Patients were followed every 1-4 months after the operation and serum tests like carcinoembryonic antigen (CEA), carbohydrate antigen 19-9 (CA19-9) and ultrasonography, spiral computed tomography, contrast magnetic resonance imaging or positron emission tomography scan when needed were utilized to exclude recurrence. Once clinically confirmed recurrence, patients would receive a second hepatic resection, transarterial catheter embolization, radiation therapy or systemic therapeutic method depended on the number, size or site of the recurrent tumor.

### Propensity Score Matching Analysis

To overcome selection bias in this observational, non-randomized study, we conducted propensity score matching (PSM) to explore the long-term survival of BTS subgroups. Logistic regression model was applied to calculate the PSM score for each individual depending on whether he had biliary tract stone or not. The covariates balanced were basic clinical features and other prognostic factors including HBV infection status, liver fluke infection status, serum γ-GGT, CEA, CA19-9, tumor number, tumor diameter, lymph node metastasis, macro-vascular invasion, micro-vascular invasion. A 1:2 matching ratio was achieved by applying the nearest neighboring algorithm with the caliper value of 0.02 to pick out the adequate matched subjects.

### Immunohistochemistry and evaluation of immunocytes and PD-L1 expression

Immunohistochemistry was conducted using GTVision^TM^III kit (Genetech, China) according to the manufacturer's instructions. Primary antibodies are as follows: CD3 (GB13014, 1:50), CD8 (GB11068, 1:200) (Servicebio, China), CD4 (YX32005, 1:200) (Wisee biotechnology, China) and CD68 (ab213363, 1:1000), PD1 (ab137132, 1:200) (Abcam, UK), PD-L1 (E1L3N, 13684, 1:1000, CST, USA). Cancerous and paired para-tumorous formalin embedded tissues from thirty ICC patients, including ten patients without BTS, ten patients with gallbladder stone and ten patients with hepatolithiasis, were stained and evaluated by two independent pathologists unaware of the group information. Three to five representative areas (200× magnification, 0.46 mm^2^ per field) of tumor or para-tumor specimens from each patient were captured by Leica DM IRE2 microscope (Leica Microsystems Imaging Solutions) for further statistical analysis. Every selected area was repeatedly analyzed for six primary antibodies list above. The PD-L1 rank was calculated using tumor proportion score (TPS) 19. Image Pro Plus 6.0 (Media Cybernetics Inc.) was utilized to count positive stained cells as previously described 20.

### Statistical analysis

Continuous variables were expressed as mean ± standard deviation and compared by Student's* t* test or Mann-Whitney* U* test for two groups and one-way ANOVA for three or more groups. Wilcoxon rank tests was used to compare categorical variables of paired groups. The cumulative OS and TTR were plotted using Kaplan-Meier method by log-rank test or Breslow-Wilcoxon test. Univariate and multivariate analyses were performed by utilizing Cox proportional hazard regression model to determine prognostic factors. A Pearson χ2 test or Fisher's exact test was used to compare categorical variables. Statistical analyses were performed by using IBM SPSS 24.0 software (SPSS) or Graphpad Prism 7.0 or R software v.3.6.1 (R Foundation for Statistical Computing, Vienna, Austria; www.r-project.org). NLR 21, PLR 22, SII 14, LMR 15, SIRI 16 were calculated as previously described. A *P* < 0.05 (two-tailed) was considered to be statistically significant.

## Results

### Description and demographics of enrolled ICC patients

**Figure 1:**
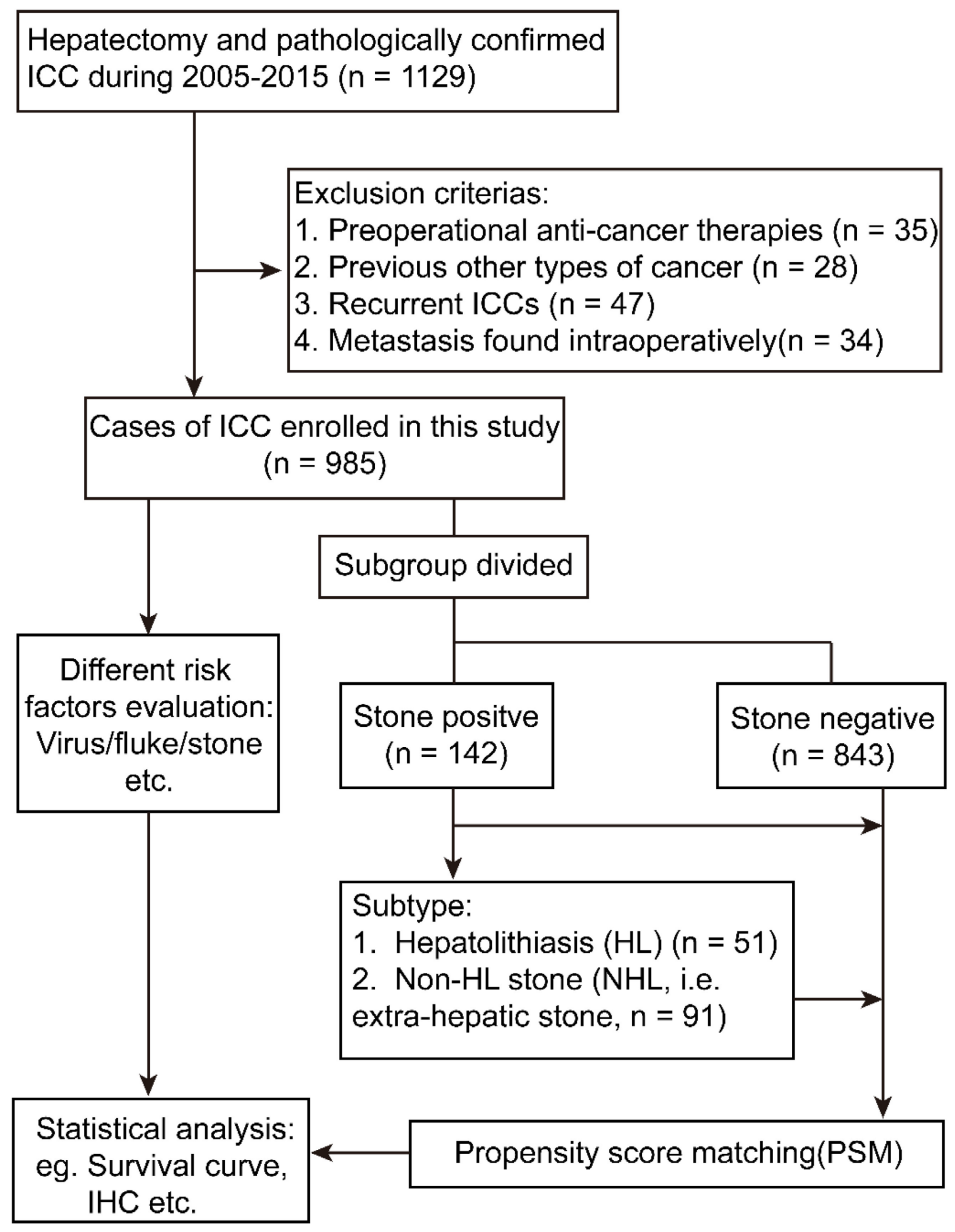
Flowchart of the present study design. ICC, intrahepatic cholangiocarcinoma; IHC, immunohistochemistry.

The analysis flow was summarized in Figure 1. Of the 985 ICC patients enrolled in this study, the median age was 58.80 ± 10.49 years, including 583 male patients and 402 female patients, and 16.9% (166/985) have multi tumors and 53.8% (530/985) have a tumor diameter which was > 5cm, and those who have lymph node metastasis occupy 16.1% (159/985). Those had macro-vessel invasion consists 5.4% (53/985) of the whole cohort. The patients diagnosed as Edmonson grade I-II and III-IV was 47.5% (468/985) and 52.5% (517/985), respectively.

### The survival indication of biliary tract stone in ICC patients before propensity score matching (PSM) analysis

We first evaluated the prognoses of ICC patients with different epidemiological risk factors and significantly different overall survival (OS) (*P* < 0.0001, Figure S1) was observed, moreover, in the first two years after hepatectomy patients with biliary tract stone seemed to have a higher mortality rate. No difference was found between different risk factor groups for time to recurrence (TTR) (*P* = 0.654, Figure S1). Of the whole ICC cohort, by using Cox hazard model we found BTS (*P* = 0.028) rather than other epidemiological factors was an independent risk factor for the OS by multivariate analysis, nonetheless, BTS was not a significant indicator for TTR (*P* = 0.209, Supplementary Table 1). Other risk factors affected OS and TTR of the cohort by Cox survival analyses were summarized in Supplementary Table 1.

**Figure 2:**
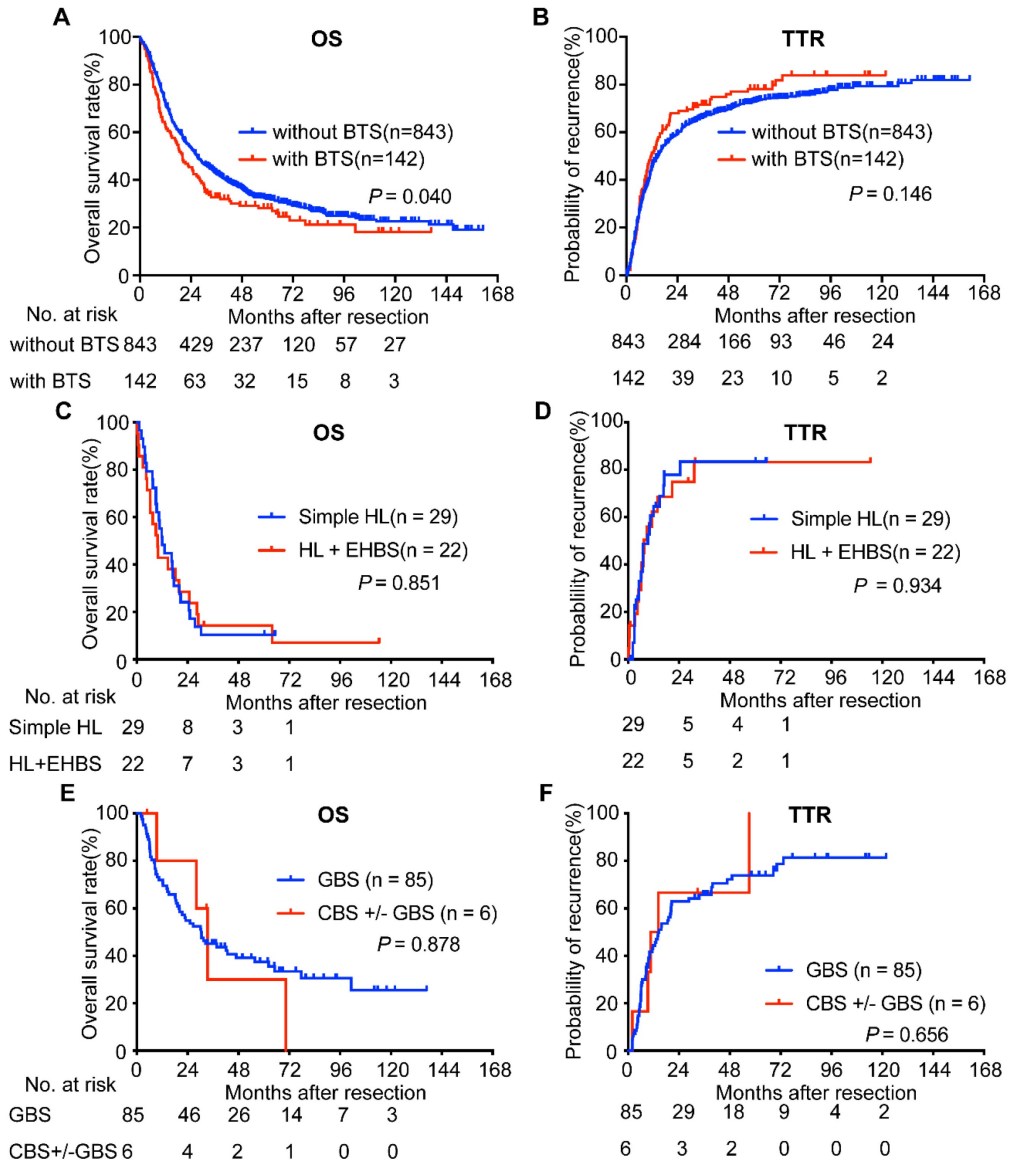
Survival analyses of ICC patients accompanied with BTS and with diverse subsets of BTS. OS (A) and TTR (B) curves of ICC patients with/without BTS; comparison of OS (C) and TTR (D) between ICC patients with simple HL and those with HL plus EHBS; analyses of OS (E) and TTR (F) between ICC patients with GBS and those with CBS accompanying GBS or not. OS, overall survival; TTR, time to recurrence. BTS, biliary tract stone; HL, hepatolithiasis; GBS, gallbladder stone; CBS, common bile duct stone; EHBS, extra-hepatic biliary stone (i.e. GBS (+) or CBS (+) or both positive)

Considering BTS as a potential survival indicator of ICC patients after surgery, we compared the survival of ICC patients with BTS and those without BTS and found the former had a worse OS (median, 20.00 vs. 26.97 months, respectively, *P* = 0.040, Figure 2A). The 1-, 3-, 5-, 7-, 9-year survival rate of subjects in BTS group and without BTS group was 63.6%, 33.7%, 29.0%, 21.8%, 18.7% and 72.8%, 43.3%, 32.6%, 27.4%, 23.8%, respectively. As for TTR, no significance was observed between enrolled subjects with BTS and those without BTS (median, 11.77 vs. 14.73 months, respectively, *P* = 0.146, Figure 2B). The 1-, 3-, 5-, 7-, 9-year recurrence rate of in no BTS group and BTS group was 55.6%, 34.3%, 27.4%, 24.8%, 20.9% and 49.8%, 29.8%, 22.9%, 16.9%, 16.9%, respectively.

We next analyzed OS and TTR of ICC patients with BTS according to the anatomic location of stone. MRI images of ICC patients with NHL or HL and without stone were retrospectively reviewed and uncovered to be hard to distinguished for their shared similar features: the lesion was hypodense on T1-weighted image but hyperdense on T2-weighted phase, and rim-like enhancement on the arterial phase and concentric filling of contrast and sustained enhancement on venous phase or delayed stage were observed in clinical practice (Figure S2). Survival analyses showed that, compared with those patients accompanying hepatolithiasis (HL) and extra-hepatic biliary stone (EHBS, i.e. GBS (+) or CBS (+) or both positive), patients with simple HL had indifferent OS (median, 9.83 months vs. 11.96 months, *P* = 0.851, Figure 2C) and TTR (median, 7.5 months vs. 9.4 months, *P* = 0.934, Figure 2D). Furthermore, comparison of OS (median, 30.45 months vs. 33.43 months, *P* = 0.878, Figure 2E) and TTR (median, 14.63 months vs. 12.5 months, *P* = 0.656, Figure 2F) between individuals with GBS and those with GBS +/- CBS didn't achieve significant difference. Therefore, we classified those with HL despite the status of EHBS as HL group, and those with GBS accompanying CBS or not as non-hepatolithiasis (NHL) group in the following analyses.

We also conducted analyses to explore whether HBV infection or liver fluke might influence the survival of ICC patients with BTS, and found OS (median, 23.10 months vs. 17.47 months, *P* = 0.293) and TTR (median, 12.73 months vs. 10.67 months, *P* = 0.885) of HBV-positive patients were indifferent to those HBV-negative patients (Figure S3A, B). Similarly, no differences of OS (median, 10.47 months vs. 20.23 months, *P* = 0.206) and TTR (median, 5.87 months vs. 12.73 months, *P* = 0.287) were found between fluke-positive patients and fluke-negative patients (Figure S3C, D).

### The survival of ICC patients with hepatolithiasis are worse than those without BTS or those with non-hepatolithiasis stone

**Figure 3:**
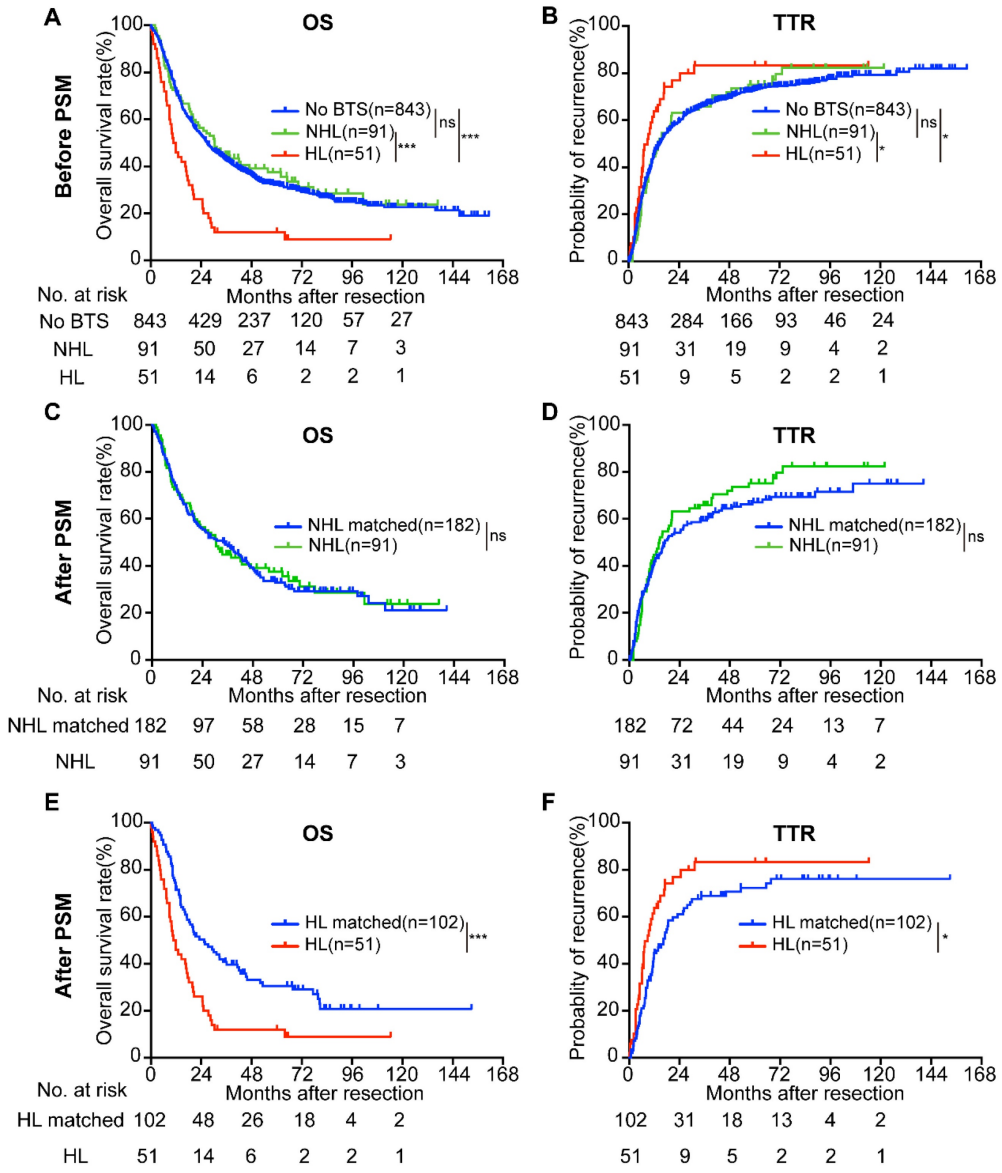
Survival analyses of enrolled ICC patients according to BTS status before and after propensity score matching (PSM) analysis. A/B, OS and TTR of ICC patients classified by BTS subtypes before PSM; C/D, survival curves of ICC patients with non-hepatolithiasis (NHL) and their matched subjects after PSM; E/F, OS and TTR compared between patients with hepatolithiasis (HL) and HL-matched group after PSM. ns, not significant; *, *P* < 0.05; ***, *P* < 0.001.

As the biliary tree consists of intra-hepatic bile duct and extra-hepatic bile duct, and stone located in different part of biliary tree may results in discrepant prognoses of ICC patients, we then divided the patients with BTS (n = 142) into two subgroups-hepatolithiasis (HL) group (n = 51) and non-hepatolithiasis (NHL) group (n = 91). KM survival analysis indicated that OS of HL group (median, 11.32 months) was inferior to no stone group (median, 26.97 months) or NHL group (median, 30.53 months) (both *P* < 0.001, Figure 3A) and TTR of no BTS group (median, 14.73 months) and NHL group (median, 14.33 months) surpassed HL group (median, 8.90 months) (*P* = 0.013 and *P* = 0.048, respectively, Figure 3B). No differences of OS and TTR between no BTS group and NHL group (*P* = 0.615 and *P* = 0.809, respectively).

Moreover, we specified impact of HBV infection on survival of patients with HL in Figure S4. Regarding ICC patients with serological HBsAg (+) or copied HBV DNA or HBeAb (+) plus HBcAb (+) as positive HBV infection, we found no differences of OS and TTR between those were HBV (+) and those were HBV (-) (median, 15.62 months vs. 7.70 months, *P* = 0.337; median, 8.2 months vs. 20.77 months, *P* = 0.328). We also found those with serological HBsAg (+) were just same individuals with copied HBV DNA and observed they had worse OS (median, 1.22 months vs. 11.90 months, *P* < 0.0001) and TTR (median, 1.22 months vs. 9.40 months; median, *P* < 0.0001) compared to those who were HBsAg (-) or HBV-DNA (-). Nonetheless, when taking statuses of HBeAb and HBcAb-markers of previous/stable HBV infection into consideration, no discrepancies of OS were exhibited between those who were serological HBeAb (+) plus HBcAb (+) (median, 10.53 months), those of HBeAb (-) plus HBcAb (+) (median, 18.20 months) and those who were HBeAb (-) plus HBcAb (-) (median, 7.70 months) (*P* = 0.185), and neither were comparison of TTR of ones were serological HBeAb (+) plus HBcAb (+) (median, 7.13 months), those of HBeAb (-) plus HBcAb (+) (median, 8.90 months) and those who were HBeAb (-) plus HBcAb (-) (median, 20.77 months) (*P* = 0.262).

As propensity score matching analysis (PSM) is a powerful tool to overcome selection bias and to increase the reliability of retrospective observational research, we performed PSM as previously described at 1:2 ratio to investigate the prognostic value of BTS, and distribution of PSM score and jitter plot of matched samples were exhibited in Figure S5 and Figure S6, respectively. Furthermore, comparison analysis exhibited a good performance and barely no difference of clinical parameters between HL group and HL-matched group was found except for CA19-9 (Table 1).

Compared with the NHL-matched group (n = 182), NHL group (n = 91) showed no significant difference in OS (median, 34.47 vs. 30.53 months, *P* = 0.878) and TTR (median, 17.10 vs. 14.33 months, *P* = 0.312) (Figure 3C, D). Nonetheless, the survival time of HL group (n = 51) was more dismal than HL-matched group (n = 102) (median, 25.57 vs. 11.32 months, *P* < 0.001 for OS; median, 16.50 vs. 8.90 months, *P* = 0.017 for TTR) (Figure 3E, F). Moreover, For the no BTS group, the multivariate regression analysis showed the risk factors for OS were CEA, CA19-9, γ-GGT, total bile acid, tumor number, tumor size, differentiation, lymph node metastasis (LNM), and for TTR were CEA, CA19-9, tumor number, tumor size, differentiation, LNM, macro-vessel invasion. The factors affected the OS of NHL stone group were direct bilirubin (DB), total bile acid, tumor number, LNM, as for TTR the significant features were tumor number, tumor size, and LNM. While the variables influencing the OS of HL group were uncovered to be CA19-9, DB and the independent risk factors for the TTR of HL group were CA19-9, total bilirubin (TB), DB and LNM (Table 2).

### Associations between preoperative peripheral inflammation parameters and ICC patients according to BTS status before/after PSM analysis

**Figure 4:**
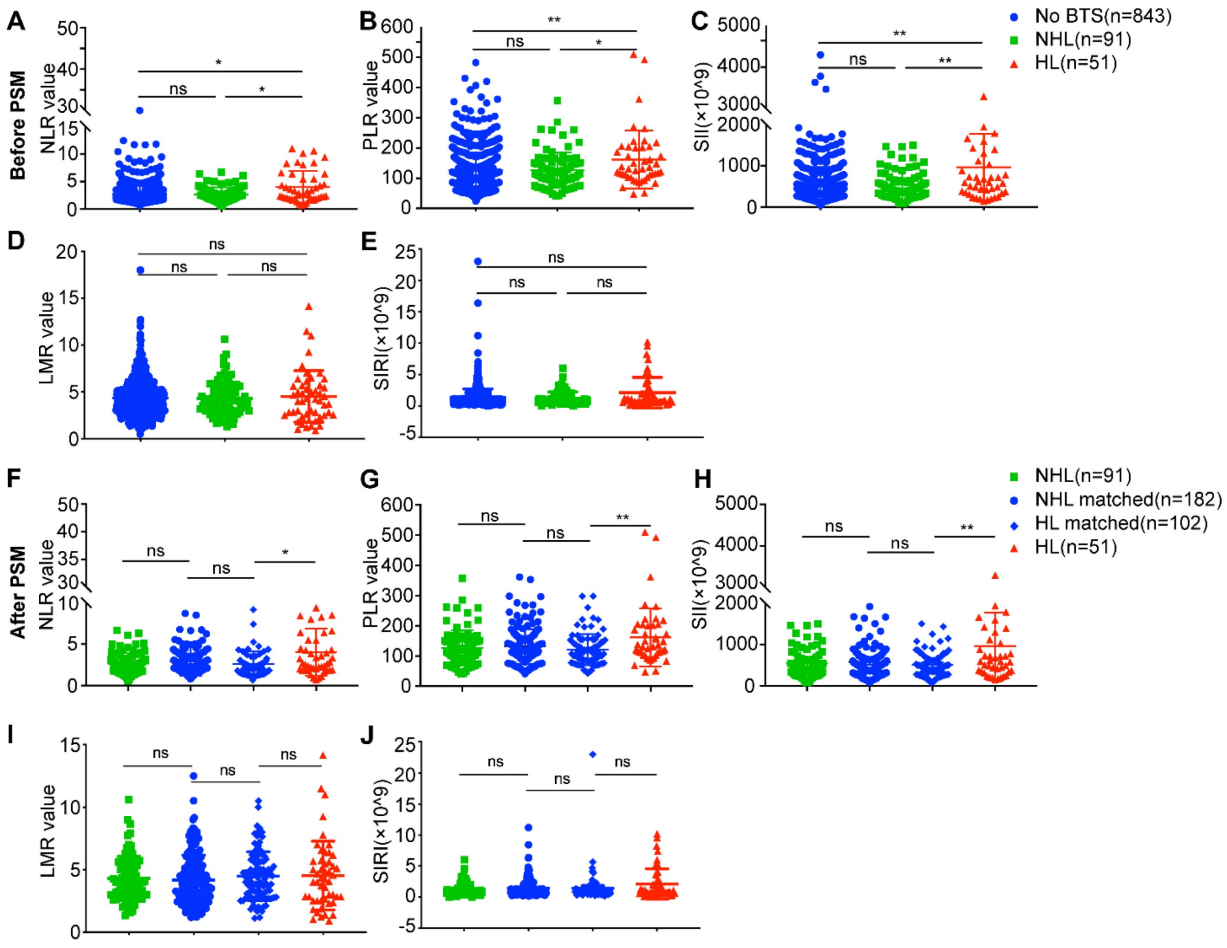
Analyses of preoperative peripheral inflammation parameters between ICC patients with diverse subsets of BTS. A/B/C/D/E, comparison of neutrophil to lymphocyte ratio (NLR)/ platelet to lymphocyte ratio (PLR)/ systemic immune inflammation (SII) values/ lymphocyte to monocyte ratio (LMR) values/ systemic inflammation response index (SIRI) values between patients with HL or with NHL and those without stone before PSM; F/G/H/I/J, comparison of NLR/PLR/SII/LMR/SIRI values between patients with HL or with NHL and their matched individuals after PSM. *, *P* < 0.05; **, *P* < 0.01, Mann-Whitney *U* test.

As a hallmark of neoplasm, inflammation is drawing fervent attention, and indices like NLR, PLR, SII, LMR and SIRI which reflect host inflammation response were reported to be of prognostic value in several solid tumors 13-16. We analyzed these indices of these three different groups, as is shown in Figure 4, the NLR values, PLR values and SII values of HL group were significantly higher than no BTS group (4.03 ± 2.86 vs. 2.82 ± 1.92, *P* = 0.019; 161.95 ± 95.71 vs. 126.31 ± 59.41, *P* = 0.004; 926.98 ± 802.39 vs. 548.11 ± 418.45, *P* = 0.001, respectively) and NHL group (4.03 ± 2.86 vs. 2.67 ± 1.26, *P* = 0.035; 161.95 ± 95.71 vs. 127.47 ± 58.48, *P* = 0.032; 926.98 ± 802.39 vs. 540.82 ± 373.29, *P* = 0.005, respectively) (Figure 4A, B, C). While no statistical significance of LMR values and SIRI values were achieved comparing no BTS group and HL group (4.33 ± 2.00 vs. 4.52 ± 2.76, *P* = 0.919; 1.30 ± 1.40 vs. 2.11 ± 2.45, *P* = 0.161, respectively), and neither were LMR values (4.29 ± 1.77 vs. 4.52 ± 2.76, *P* = 0.860) and SIRI values (1.23 ± 1.00 vs. 2.11 ± 2.45, *P* = 0.111) between NHL group and HL group (Figure 4D, E). No differences of NLR values (*P* = 0.886), PLR values (*P* = 0.757), SII values (*P* = 0.729), LMR values (*P* = 0.993) and SIRI values (*P* = 0.548) were observed between no BTS group and NHL group.

After PSM analysis, NLR values, PLR values and SII values of HL group remained higher than HL-matched group (4.03 ± 2.86 vs. 2.60 ± 1.51, *P* = 0.015; 161.95 ± 95.71 vs. 121.34 ± 51.52, *P* = 0.005; 926.98 ± 802.39 vs. 500.91 ± 291.96, *P* = 0.003, respectively) (Figure 4F, G, H), however, LMR values (4.52 ± 2.76 vs. 4.48 ± 1.95, *P* = 0.618) and SIRI values (2.11 ± 2.45 vs. 1.40 ± 2.39, *P* = 0.148) of HL group showed to be indifferent to HL-matched group (Figure 4 I, J). Besides, there existed no differences of NLR values, PLR values, SII values, LMR values and SIRI values between NHL group and NHL-matched group or between HL-matched group (all *P* > 0.05). The above results indicated unique immune response may to some extent contribute to unfavorable prognoses of HL-related ICC.

### Comparison of tumor infiltrating immunocytes, PD1^+^ cells and PD-L1 levels in different subgroups of ICC patients

**Figure 5:**
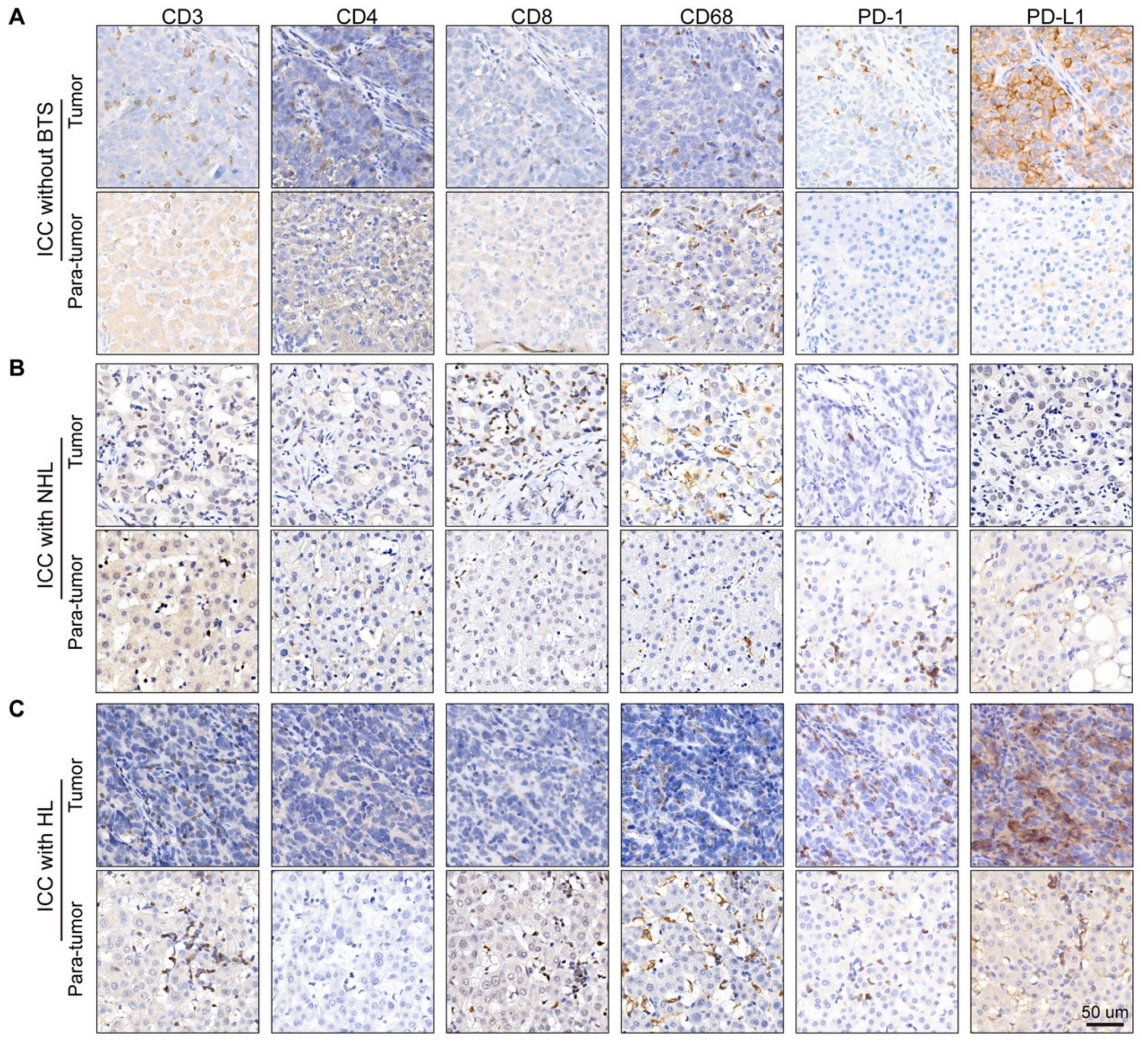
Staining of immunocyte markers in ICC patients without BTS, with NHL and with HL. A/B/C, typical staining of CD3, CD4, CD8, CD68, PD1 and PD-L1 in tumorous and para-tumorous tissues of ICC patients without BTS, with NHL and with HL, respectively.

Owing to the previous results, we hypothesize immune response may have a role in the progression of stone-related ICC. Then, we conducted immunostaining of CD3 (pan-T cell marker), CD4 (T-helper cell marker), CD8 (cytotoxic T cell marker), CD68 (macrophage marker) and PD1/PD-L1 (immune checkpoint protein) in tumor specimen and paired para-tumor tissue of ten ICC patients without BTS, ten ICC patients with NHL stone and ten ICC patients with HL (Figure 5A/B/C). Densities of immunocytes varied vastly interindividually, even in one gross formalin-fixed sample section the immunocytes distribution appeared to be remarkably heterogeneous. Moreover, immunocytes of adjacent liver tissues infiltrated mainly in the portal areas and generally the denseness of immune cells in tumorous tissues was inferior to those in paired para-tumor specimens.

**Figure 6:**
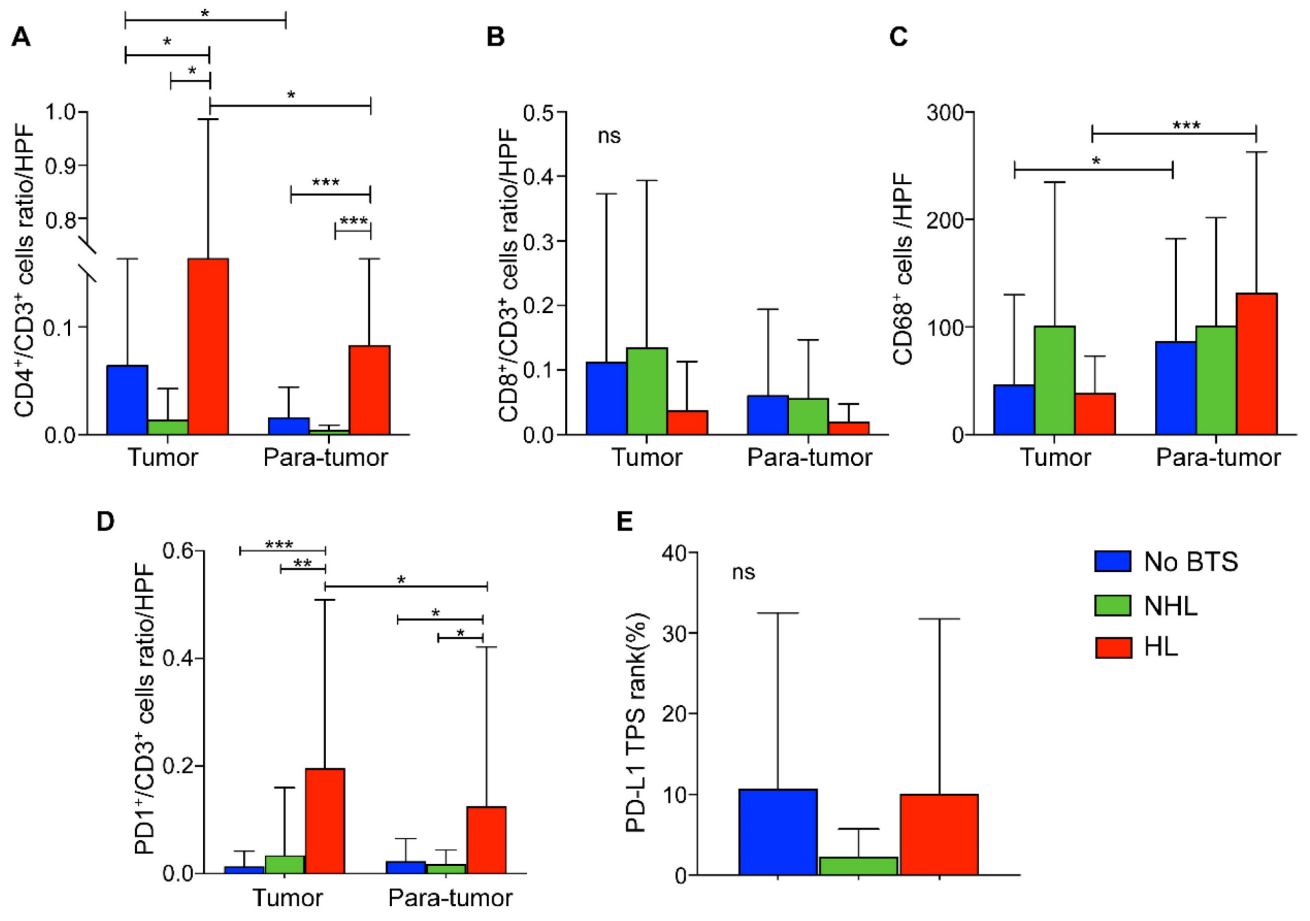
Comparison of immunocyte ratios/numbers and PD-L1 level in ICC without BTS, with NHL and with HL. A, CD4^+^/CD3^+^ cells ratio per HPF among subgroups; B, CD8^+^/CD3^+^ cells ratio per HPF among subgroups; C. CD68^+^ macrophages per HPF among subgroups; D. PD1^+^/CD3^+^ cells per HPF among subgroups; E, PD-L1 rank among subgroups. ns, not significant; *, *P* < 0.05; **, *P* < 0.01; ***, *P* < 0.001, one-way ANOVA test. HPF, high power field.

Compared with HL group, tumorous CD4^+^/CD3^+^ T lymphocytes ratios per high power field (HPF) in NHL group and no BTS group were significantly lower (*P* = 0.015 and 0.036, respectively). Similarly, CD4^+^/CD3^+^ T lymphocytes ratio of HL group was higher in para-tumor tissue than the other two groups (both* P* < 0.001, Figure 6A). Besides, CD4^+^/CD3^+^ cells ratio in cancerous tissues of HL group surpassed those in the paired adjacent tissues (*P* = 0.048, Figure 6A). Though CD8^+^/CD3^+^ T cell ratios per HPF of HL group seemed to be lowest in tumor and para-tumor specimen, no significant difference was detected among those three groups (all *P* > 0.05, Figure 6B). Moreover, CD68^+^ macrophages infiltrated in para-tumorous specimen of HL group and no BTS group significantly exceeded those in paired cancerous samples (*P* < 0.001 and 0.015, respectively, Figure 6C) and though it did not reach statistically significance there existed a growing tendency of para-tumorous CD68^+^ cell numbers from no BTS group, NHL group to HL group (*P* = 0.201).

Of note, PD1^+^/CD3^+^ T lymphocytes ratio per HPF in tumor samples of HL group outnumbered that in adjacent liver tissues (*P* = 0.017), and the ratio was also found higher in tumorous tissues and para-tumor samples of HL group than those in no BTS group (*P* < 0.001 and *P* = 0.024, respectively) or NHL group (*P* = 0.002 and 0.029, respectively, Figure 6D). Besides, no discrepancy of PD-L1 TPS rank between no BTS group, NHL group and HL group were observed (*P* = 0.580, Figure 6E).

### Correlations of preoperative peripheral inflammation parameters and tumor infiltrating immunocytes and PD-L1 expression

**Figure 7:**
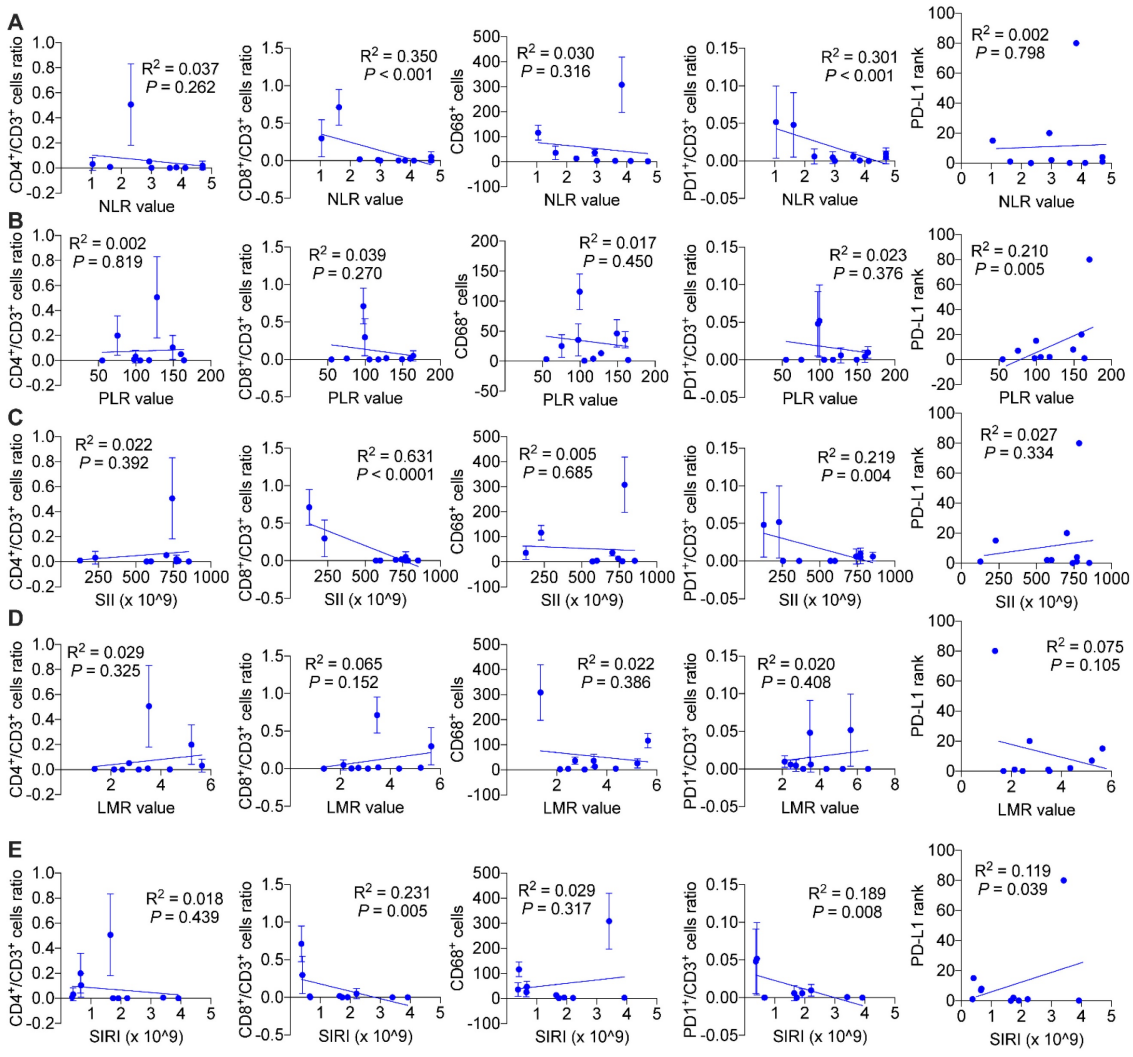
Associations of preoperative peripheral inflammation parameters and tumorous immunocyte ratios/numbers and PD-L1 rank of no stone group. Correlogram of NLR values (A), PLR values (B), SII values (C), LMR values (D) and SIRI values (E) with immunocyte ratios/numbers and PD-L1 expression by simple linear regression analyses

**Figure 8:**
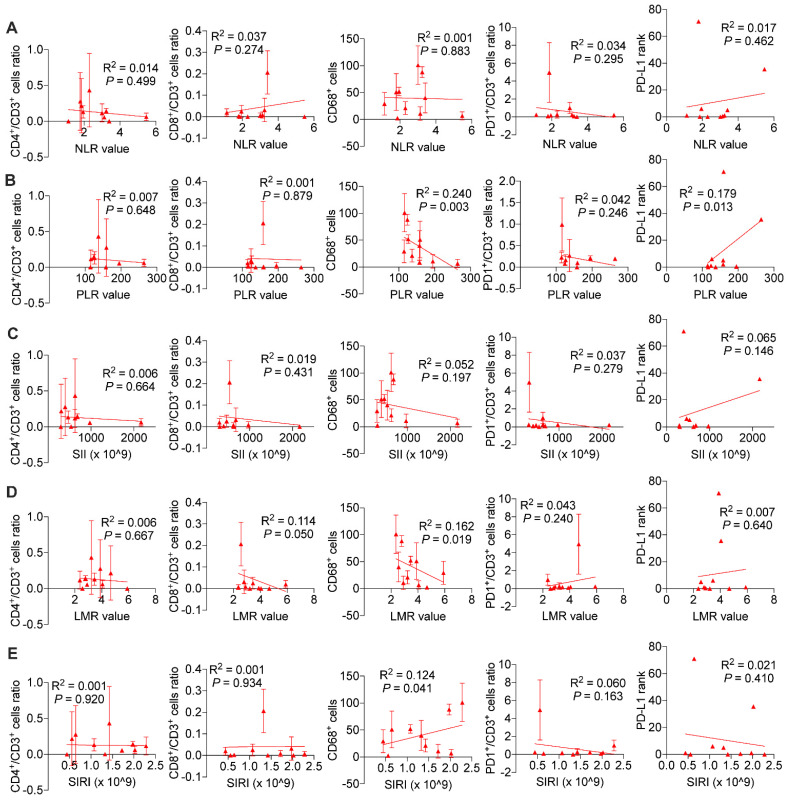
Associations of preoperative peripheral inflammation parameters and tumorous immunocyte ratios/numbers and PD-L1 rank of HL group. Correlogram of NLR values (A), PLR values (B), SII values (C), LMR values (D) and SIRI values (E) with immunocyte ratios/numbers and PD-L1 expression by simple linear regression analyses

We wondered and explored whether systemic immune response might influence local tumor immune environment. As is shown in Figure 7, we found in ICC patients without BTS, NLR values (*P* < 0.001 and *P* < 0.001, respectively), SII values (*P* < 0.001 and *P* = 0.004, respectively), SIRI values (*P* = 0.005 and *P* = 0.008, respectively) were negatively associated with CD8^+^/CD3^+^ lymphocytes ratio and PD1^+^/CD3^+^ lymphocytes ratio, and PLR values (*P* = 0.005) and SIRI values (*P* = 0.039) were positively correlated with PD-L1 levels. While in patients with NHL, NLR values had a negative relationship with CD4^+^/CD3^+^ lymphocytes ratio (*P* = 0.003) and CD68^+^ macrophages (*P* = 0.033). PLR values (*P* = 0.016 and *P* = 0.004, respectively) and SII values (*P* = 0.005 and *P* = 0.002, respectively) both were positively correlated with CD8^+^/CD3^+^ lymphocytes ratio and PD1^+^/CD3^+^ lymphocytes ratio. LMR values had a positive association with CD4^+^/CD3^+^ lymphocytes ratio (*P* = 0.001) and CD8^+^/CD3^+^ lymphocytes ratio (*P* = 0.001), while SIRI values were negatively related with CD8^+^/CD3^+^ lymphocytes ratio (*P* = 0.025) and CD68^+^ macrophages (*P* = 0.011). Besides, SII values were negatively associated with PD-L1 level (*P* = 0.010) (Figure S7). As for HL group, CD68^+^ macrophage numbers were exhibited to be negatively pertinent with PLR values (*P* = 0.003) and LMR values (*P* = 0.019), but positively correlated with SIRI values (*P* = 0.041). There existed a positive relationship between PLR values and PD-L1 expression (*P* = 0.013) (Figure 8).

## Discussion

Mounting attraction is drawn to ICC due to its rising morbidity worldwide. The present study focused on the effect of different epidemiologic risk factors on the long-term survival of a large-scale ICC cohort and investigated holistic status of immune response and locally infiltrated immunocytes in different subtypes of BTS-related ICC to enrich understandings and to prompt potential new cures for this malignancy.

Firstly, we explored the impact of different risk factors on the prognoses of the studied ICC cohort. The survival analyses indicated BTS group had a worse OS but no discrepant TTR compared with other groups. Though hepatolithiasis (HL) was documented as an unfavorable prognostic factor of ICC by several studies 23, 24, the prognostic role of extra-hepatic biliary stone in ICC and the underlying mechanism of poor survival of HL-related ICC patients had not been elucidated. We detailedly verified that ICC patients with HL, accompanying EHBS or not, suffered with shorter survival time than those without BTS or those with NHL.

Hepatolithiasis is mainly cholesterol-rich brown pigment stone, suggesting possibly similar causes with cholesterol gallbladder stone such as underlying metabolic defects, and contains more cholesterol and less calcium bilirubinate than brown stones in the common bile duct 25. We uncovered that HL-related ICC patients had the worst survival which was consistent with findings from Wang *et al.* 23 and Zhang *et al.* 24, even after covariates like HBV infection status, liver fluke infection and other factors were matched. However, they just focused on the effect of HBV infection and intrahepatic stone on patients' survival and didn't take liver fluke into analysis and didn't explore influence of HBV infection on survival of BTS-related or HL-related ICC patients. In Figure S3, we analyzed and uncovered HBV infection and liver fluke failed to contribute to poorer prognoses of BTS-related ICC patients. We also noticed that serological HBsAg (+) aggravated dismal survival, but considering statuses of HBeAb and HBcAb indicating chronic HBV infection, no discrepancies were exhibited among different groups of HL-related ICC individuals in Figure S4, which might be partly explained by HBV infection changed local immune microenvironment of the liver^26^ and anti-HBV drug might exert a positive influence on patients' survival^27^. Furthermore, our results revealed that no divergent survival between extra-hepatic biliary stone group (NHL group) and no BTS group, which differs with the viewpoint that bile duct stones are important risk factors from the meta-analysis performed by Cai *et al*.^6^, and the reason may be that the meta-analysis just included case-control studies which focused on ICC patients and their tumor-free controls and association analysis but not survival comparison was carried out.

Being a hallmark of neoplasm 8, tumor-promoting inflammation is now drawing rising attention. Preoperative peripheral inflammation parameters (PPIP) like NLR, PLR, SII, LMR and SIRI-indicators of systemic inflammation reaction-were reported to reflect survival of patients with solid tumors, which was believed to be induced by cytokine cascades released during reciprocal interaction of tumor cells and immune cells. In this study we uncovered the NLR, PLR, and SII values but not LMR and SIRI values were significantly higher in HL group compared with NHL group or no BTS group even after PSM analysis. Besides, we uncovered that though PPIP could not reflect tumorous infiltration of immunocytes, there did exist positive correlations between PPIP and immunocyte ratio and the correlations varied among subgroups according to BTS status. Neutrophils were reported to produce cytokines like VEGFA which promotes tumor angiogenesis and tends to suppress lymphocytes-mediated cytolysis of cancer cell 28. Neutrophils also help to promote immunosuppressive environment by induce Treg differentiation 29 and apoptosis of CD8^+^ T lymphocytes 30. Peripheral platelet augmentation induced by neoplasm was revealed to exert a protective effect to keep tumor cells from killing by NK cells 31. Moreover, CCR2+ monocytes recruited by tumor cells from blood lead to abundance of M2-like macrophages and exhaustion of CD8^+^ T cells by secreting CCL2^32^. Though further researched are needed to explore to what extent PPIP might influence local infiltration of immunocytes, the present data hinted us the systemic immune response was hyper-activated in HL-related ICC patients.

Luis E *et al.* discovered that immune-mediated process like neutrophil extracellular traps was indispensable for the formation of gallstone 18, which further arouse our interest on tumor immune microenvironment of BTS-related ICCs. We hypothesized that tumor infiltrating immunocytes may vary among BTS subgroups of the studied cohort considering their holistic immune status being different. Conventionally, CD4^+^ T lymphocytes relay tumor antigen to cytotoxic CD8^+^ T cells to exert antitumor effects, but the process was often hampered by regulatory T cells (Treg) consisting up to 50% of CD4^+^ T lymphocytes through establishing an immunosuppressive environment 33. In renal cancer, high tumorous infiltration of CD4^+^ T cells indicated a poorer recurrence-free survival 34. Likely, elevated stromal CD4^+^ T cell level in pancreatic neuroendocrine tumor correlated with shorter recurrence-free survival 35. Our results indicated tumoral CD4^+^/CD3^+^ T cells ratio was highest in HL group which might possibly due to augmented Treg proportion of whole CD4^+^ T cell population and further study are warranted. Moreover, CD4^+^ T lymphocytes exhibited direct eradication ability of tumor cells by IL2 neutralization 36 which makes targeting CD4^+^ T lymphocytes a promising therapy for HL-related ICC. Though not reaching statistically significance, we did observe a reduction of CD8^+^ T cells ratio in HL group which partly assist to deepen the understanding of poor prognoses of HL-related ICC.

Macrophages exhibit fickle roles in malignancies due to flexible polarization status induced by various stimuli. Hasita *et al.* observed lower peri-tumoral macrophage density correlated with a poorer postoperative survival in ICC 37, nonetheless, Oishi *et al.* reported a contrary result that enriched tumor-peripheral macrophages endowed dismal survival of ICC patients 38. Moreover, Zhu *et al.* proposed CD68^+^ macrophages enriched in para-tumor specimens produced mileus suitable for intrahepatic metastasis and forecasted poorer prognoses of HCC patients 39. Our results verified peri-tumoral infiltration level of CD68^+^ macrophages compared to paired tumor samples of no BTS group and HL group, indicating macrophage-based therapeutics like CD47 blockade that promotes macrophage phagocytosis 40 being possibly feasible. Additionally, we stained PD1 and PD-L1-vital checkpoint protein in immune escape and found relatively higher tumorous infiltration of PD1^+^/CD3^+^ cells ratio in HL-related ICC patients which indicates PD1 blockade that reverse exhausted status and then restore surveillance role of T lymphocytes 41 might be promising. Additionally, no significance of PD-L1 level were observed among studied ICC subgroups according to BTS status, varying from results from Shi *et al.* who reported lower PD-L1 expression in tumorous tissue of ICC patients with HL compared with HBV-positive ICC patients 42, the possible reasons might lie in the distinct grouping method and small sample sizes investigated in both two studies.

The present research is highlighted as follows: (1) it for the first time explored the impact of intra-hepatic (HL group) and extra-hepatic biliary stone (NHL group) on the long-term prognoses (up to 14 years) of a large-scale ICC cohort and found HL group, rather than NHL group or no BTS group, possessed the worst survival even after PSM analysis; (2) systemic immune reaction was hyper-activated in HL group compared with NHL group or no BTS group; (3) targeting T helper or cytotoxic T cell in the tumorous tissue and macrophages in the peri-tumor tissues are possibly effective treatments for HL-related ICC. Nonetheless, the limitations should be noted: the present study was retrospectively observational and based on single-center follow-up data and caution should be warranted to interpret the distribution of immunocytes considering the small sample set. Further prospective interventional approaches targeting host immune system are needed.

In summary, this study reported hepatolithiasis instead of extra-hepatic biliary stone was a poor survival indicator for ICC patients received cure-intention hepatectomy and suggested a role of systemic and local immune response might contribute to dismal survival and proposed the possibility of immunotherapy as potential treatments for HL-related ICC patients.

## Supplementary Material

Supplementary figures and table.

## Figures and Tables

**Table 1:** Clinical characteristics of ICC patients without biliary tract stone (BTS) and those with hepatolithiasis (HL) before and after PSM analysis

Variable	Before PSM			After PSM		
	Without BTS	HL	*P* value	HL-matched	HL	*P* value
	(n = 843)	(n = 51)		(n = 102)	(n = 51)	
Age			.944			.909
< 60	434	26		51	26	
≥ 60	409	25		51	25	
Gender			.309			.645
female	336	24		44	24	
male	507	27		58	27	
HBV			.103			.386
positive	632	33		73	33	
negative	211	18		29	18	
Fluke			1.000^a^			1.000^a^
positive	57	3		5	3	
negative	786	48		97	48	
TB, umol/L	21.95±51.34	29.80±58.21	.308	30.06±68.81	29.80±58.21	.982
DB, umol/L	11.99±37.09	20.15±49.18	.269	15.84±45.87	20.15±49.18	.614
ALB, g/L	41.52±3.83	40.44±4.20	.064	40.79±3.93	40.44±4.20	.636
TBA, umol/L	16.65±45.13	22.89±62.01	.369	24.29±67.36	22.89±62.01	.906
ALT, U/L	49.16±127.39	35.70±31.46	.466	43.54±55.98	35.70±31.46	.372
AST, U/L	49.26±153.47	33.98±25.15	.491	40.19±47.00	33.98±25.15	.396
γ-GGT, U/L	133.07±247.36	292.29±368.51	.005	181.94±471.51	292.29±368.51	.163
Prealbumin, g/L	0.23±0.06	0.21±0.06	.146	0.22±0.06	0.21±0.06	.664
AFP, ng/ml	68.48±756.08	2.70±2.99	.547	239.06±2045.60	2.70±2.99	.426
CEA, ng/ml	18.67±210.52	19.54±39.28	.978	8.86±23.47	19.54±39.28	.096
CA19-9, U/ml	812.66±2228.60	2525.65±3403.84	.002	986.78±2208.49	2525.65±3403.84	.007
Tumor Number			.091			1.000^a^
single	701	47		94	47	
multiple	142	4		8	4	
Tumor size			.617			.137
≤ 5cm	394	22		57	22	
> 5cm	449	29		45	29	
Encapsulation			.279			.455
absence	733	47		90	47	
present	110	4		12	4	
LNM			.207			.716
positive	139	5		12	5	
negative	704	46		90	46	
Macro-VI			1.000^a^			1.000^a^
yes	45	3		5	3	
no	798	48		97	48	
Differentiation			.157			.067
I-II	400	19		54	19	
III-IV	443	32		48	32	
BCLC stage			.871			.489
0+A	321	20		46	20	
B+C	522	31		56	31	
TNM^b^			.822			.783
0-II	656	39		80	39	
III	187	12		22	12	

TB, total bilirubin; DB, direct bilirubin; ALB, albumin; TBA, total bile acid, ALT, alanine aminotransferase; AST, aspartic aminotransferase; γ-GGT, gamma-glutamyl transferase; CEA, carcinoembryonic antigen; CA19-9, carbohydrate antigen 19-9; Macro-VI, macrovascular invasion; Micro-VI, microvascular invasion BCLC, Barcelona clinical liver cancer stage a, Chi-Square continuity correction b, TNM, the AJCC 8th edition

**Table 2:** Multivariate survival analyses of ICC patients without biliary tract stone (BTS) or non-HL stone or hepatolithiasis by Cox proportional hazard model

Variable	OS			TTR	
	HR (95%CI)	*P*		HR (95%CI)	*P*
**Without BTS (n = 843)**					
TB, umol/L	/	/		0.997(0.994-1.000)	.087
CEA, ng/ml	1.000(1.000-1.001)	.016		1.000(1.000-1.001)	.023
CA19-9, U/ml	1.000(1.000-1.000)	< .001		1.000(1.000-1.000)	< .001
Total bile acid, umol/L	1.003(1.001-1.005)	.001		/	/
Tumor Number (multiple vs. single)	1.535(1.214-1.942)	< .001		1.555(1.234-1.961)	< .001
Tumor size (> 5cm vs. ≤ 5cm)	1.553(1.276-1.892)	< .001		1.559(1.284-1.894)	< .001
Differentiation (III-IV vs. I-II)	1.403(1.163-1.691)	< .001		1.365(1.134-1.643)	.001
LNM (yes vs. no)	2.320(1.839-2.926)	< .001		1.812(1.419-2.315)	< .001
Macro-vessel invasion (yes vs. no)	1.396(0.974-2.000)	.069		1.615(1.107-2.355)	.013
**NHL stone (n = 91)**					
DB, umol/L	1.020(1.008-1.032)	.001		/	/
Total bile acid, umol/L	0.986(0.973-0.998)	.027		/	/
Tumor Number (multiple vs. single)	3.529(1.867-6.672)	< .001		2.120(1.202-3.738)	.002
Tumor size (>5 cm vs. ≤ 5 cm)	/	/		2.120(1.202-3.738)	.009
LNM (yes vs. no)	7.223(3.442-15.157)	< .001		10.256(4.254-23.253)	< .001
**Hepatolithiasis (HL, n = 51)**					
CA19-9, U/ml	1.000(1.000-1.000)	.011		1.000(1.000-1.000)	.003
TB, umol/L	/	/		0.828(0.711-0.964)	.015
DB, umol/L	1.010(1.001-1.020)	.039		1.245 (1.050-1.475)	.011
LNM (yes vs. no)	/	/		4.513(1.268-13.602)	.019
