# Supplementary material for: Evaluating prognostic value of biliary stone in intrahepatic cholangiocarcinoma by propensity score matching analysis
Source: J Cancer. 2023 May 8;14(7):1257–71. doi: 10.7150/jca.74275 (PMC10197931; doi:10.7150/jca.74275)
Supplement: Supplementary file 1 — Supplementary figures and table. [file jcav14p1257s1.pdf]

Supplementary Figures and Tables

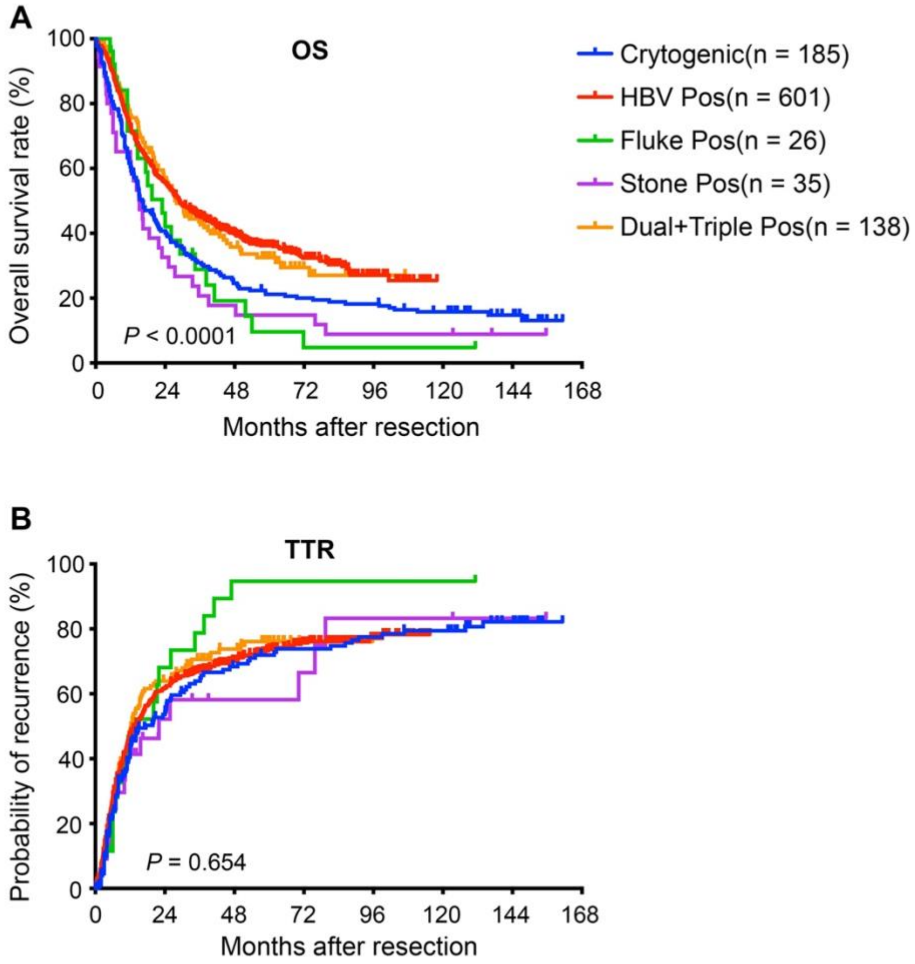

Figure S1 Overall survival (OS) and time to recurrence (TTR) of enrolled ICC patients grouped by different risk factors. A, OS of various risk factor groups; B, TTR of various risk factor groups. Pos, positive.

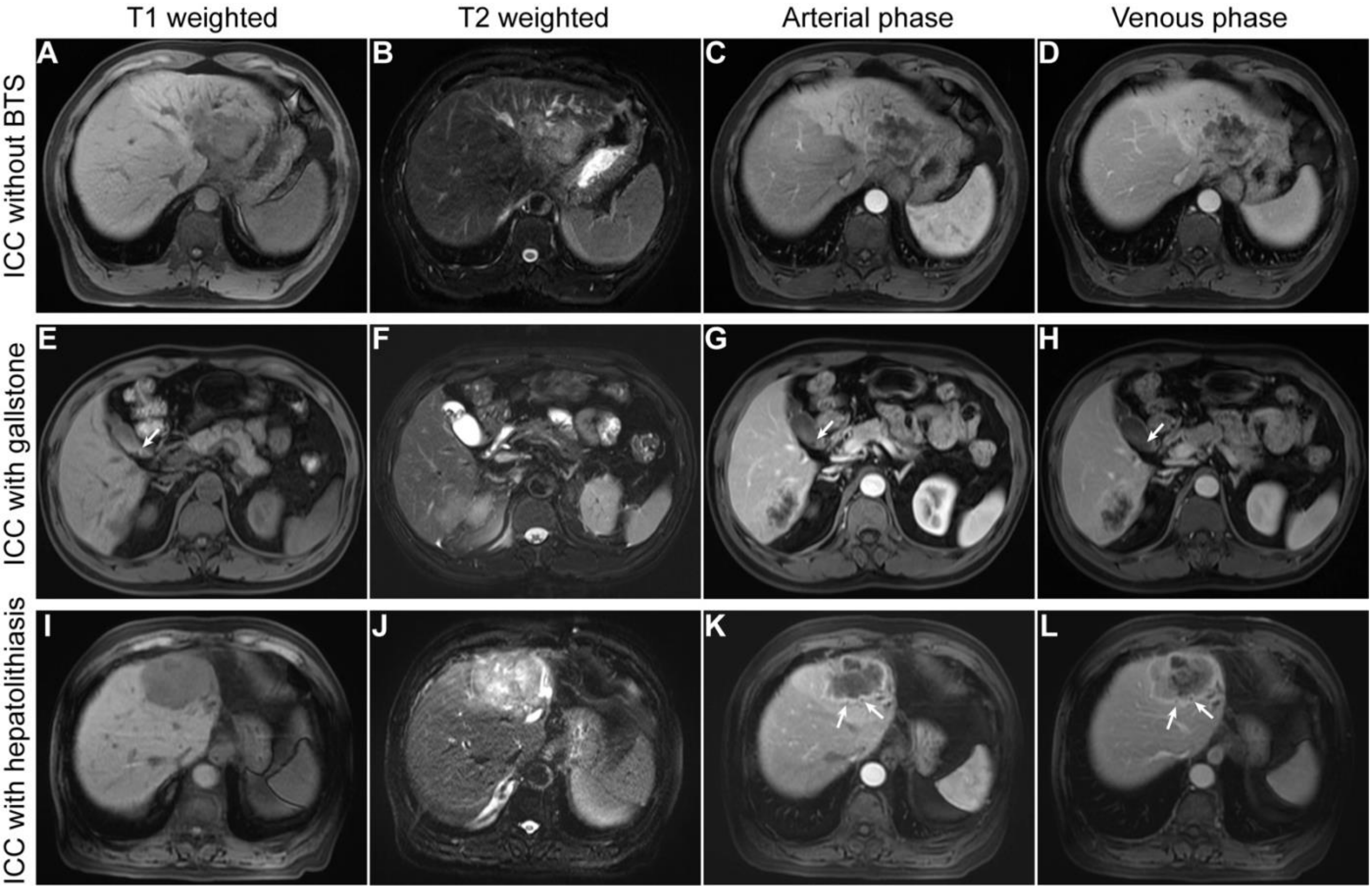

Figure S2 Typical MRI pictures of ICC patients accompanying with or without biliary tract stones (BTS). A/E/I, T1-weighted images; B/F/J, T2-weighted images; C/G/K, arterial phase images; D/H/L, venous phase images. White arrow, pointing biliary stone.

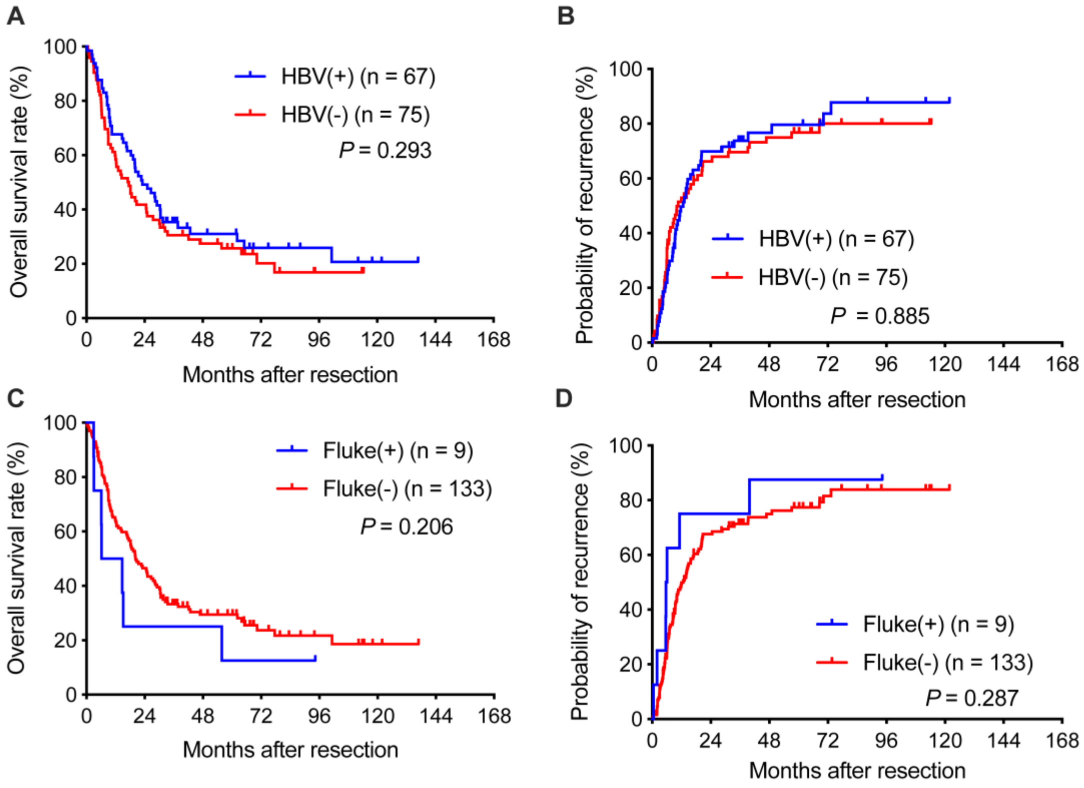

Figure S3 Impact of HBV infection or liver fluke on the prognoses of BTS-related ICC patients. OS curves of BTS-related ICC patients with/without HBV infection (A) and with/without fluke (C); TTR curves of BTS-related ICC patients with/without HBV infection (B) and with/without fluke (D)

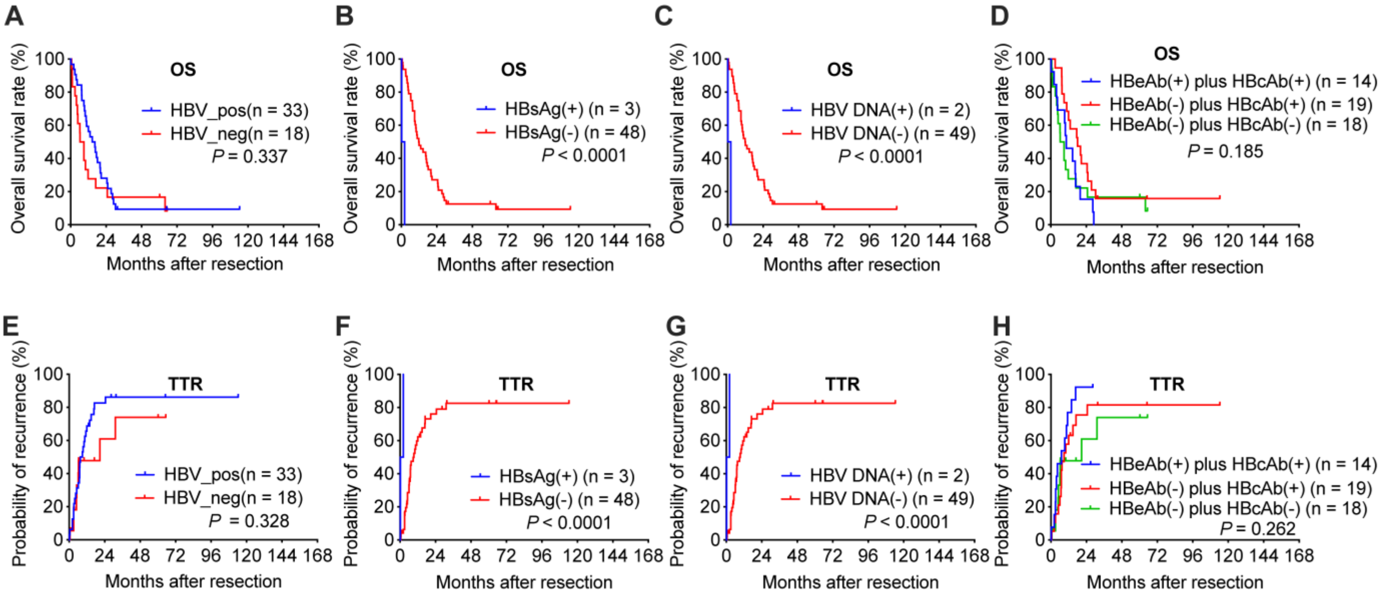

Figure S4 Impact of specific HBV serological tests on prognoses of HL-related ICC. Comparisons of OS time of HL-related ICC patients with HBV infection (pos vs. neg, A), HBsAg test (pos vs. neg, B), HBV DNA copies (pos vs. neg, C), HBeAb and HBcAb status (D); TTR curves of HL-related ICC patients with HBV infection (pos vs. neg, E), HBsAg test (pos vs. neg, F), HBV DNA copies (pos vs. neg, G), HBeAb and HBcAb status (H). Those who were serological HBsAg (+) or HBV DNA copies (+) or HBeAb (+) plus HBcAb (+) were defined as HBV positive.

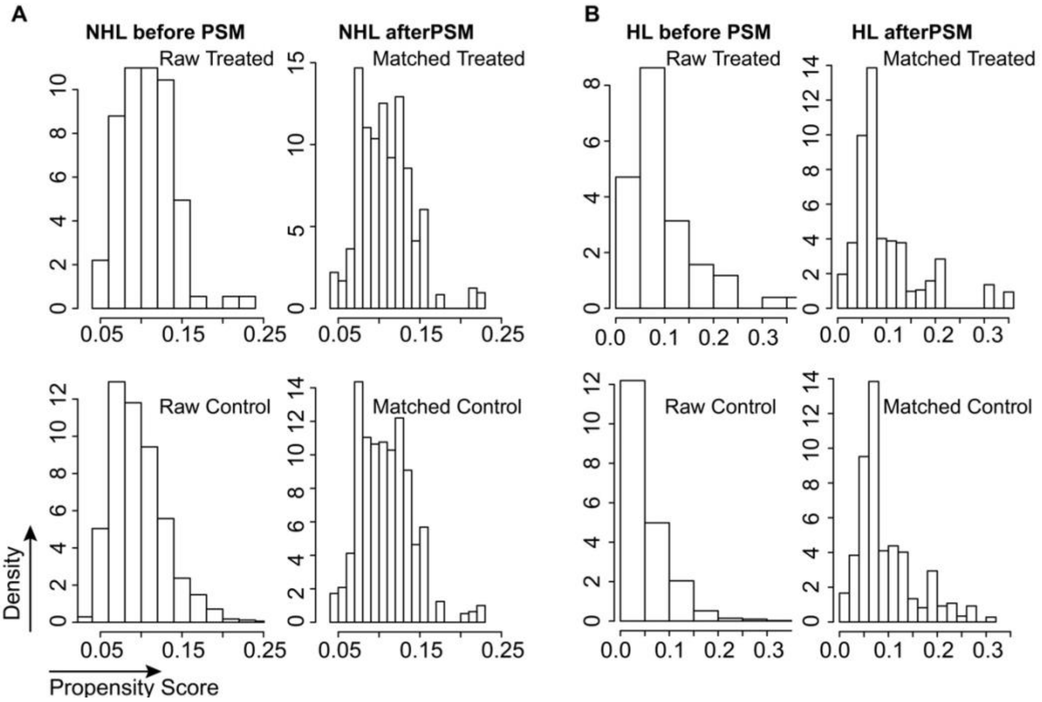

Figure S5 Distribution histograms of propensity scores of NHL group (A) and HL group (B) and their matched samples before and after PSM analyses

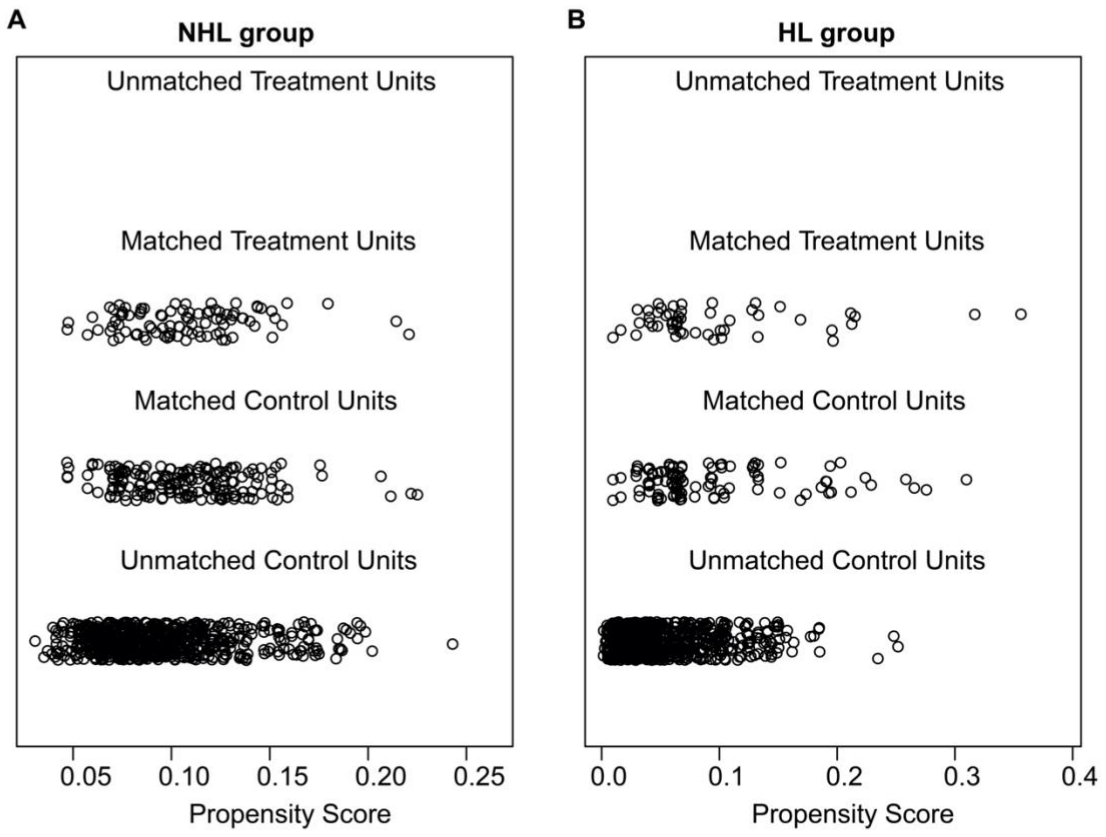

Figure S6 Jitter plots exhibited NHL group (A) and HL group (B) were generally matched at 1:2 ratio by PSM analyses

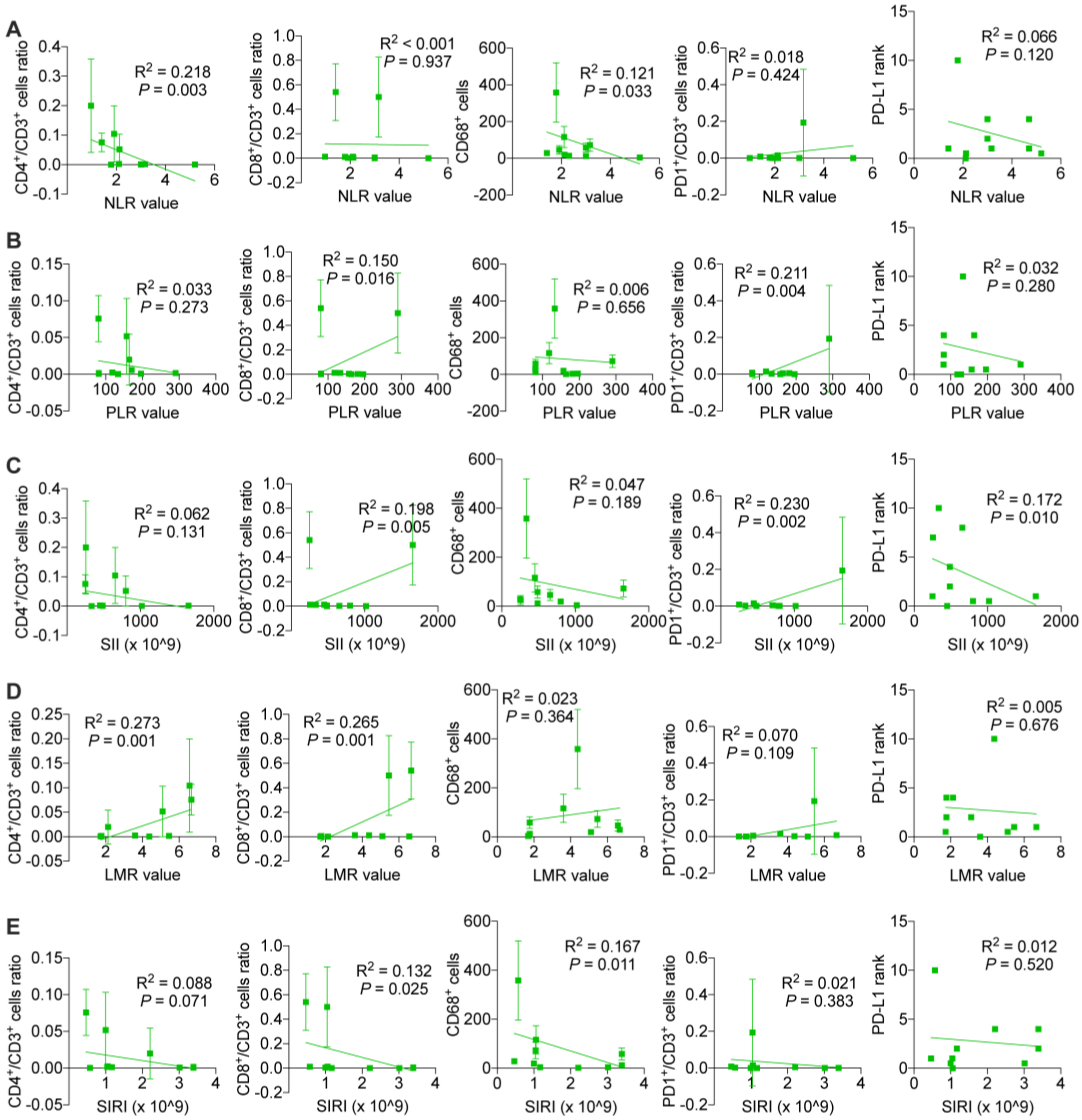

Figure S7 Associations of preoperative peripheral inflammation parameters and tumorous immunocyte ratios/numbers and PD-L1 rank of NHL group. Correlogram of NLR values (A), PLR values (B), SII values (C), LMR values (D) and SIRI values (E) with immunocyte ratios/numbers and PD-L1 expression by simple linear regression analyses

Supplementary Table 1 Univariate and multivariate survival analyses of the studied ICC cohort by Cox proportional hazard model

Variable	OS				TTR			
	Univariate		Multivariate		Univariate		Multivariate	
	HR(95%CI)	*P*	HR(95%CI)	*P*	HR(95%CI)	*P*	HR(95%CI)	*P*
Age (≥ 60 vs. < 60)	1.114(0.957-1.297)	.162			0.971(0.833-1.131)	.704		
Gender (male vs. female)	1.107(0.948-1.293)	.197			1.103(0.944-1.289)	.218		
HBV (pos vs. neg)	0.756(0.639-0.893)	**.001**	/	/	0.980(0.821-1.170)	.824		
Fluke (pos vs. neg)	1.231(0.921-1.647)	.161			1.051(0.772-1.432)	.751		
Stone (pos vs. neg)	1.235(1.001-1.524)	**.049**	1.291(1.028-1.622)	**.028**	1.146(0.926-1.419)	.209	/	/
AFP, ng/ml	1.000(1.000-1.000)	.436			1.000(1.000-1.000)	.123		
CEA, ng/ml	1.000(1.000-1.001)	**.001**	/	/	1.000(1.000-1.001)	**.002**	1.000(1.000-1.001)	**.030**
CA19-9, U/ml	1.000(1.000-1.000)	< **.001**	1.000(1.000-1.000)	< **.001**	1.000(1.000-1.000)	< **.001**	1.000(1.000-1.000)	< **.001**
TB, umol/L	1.004(1.003-1.005)	< **.001**	/	/	1.000(0.997-1.002)	.670		
DB, umol/L	1.006(1.004-1.008)	< **.001**	/	/	1.000(0.997-1.003)	.897		
Total bile acid	1.004(1.002-1.005)	< **.001**	/	/	1.000(0.998-1.002)	.766		
Albumin, g/L	0.945(0.925-0.965)	< **.001**	0.959(0.937-0.980)	< **.001**	0.977(0.957-0.998)	**.029**	/	/
γ-GGT, U/L	1.001(1.001-1.001)	< **.001**	1.001(1.000-1.001)	< **.001**	1.001(1.000-1.001)	< **.001**	/	/
Tumor Number (multiple vs. single)	1.762(1.453-2.136)	< **.001**	1.722(1.378-2.152)	< **.001**	1.910(1.577-2.314)	< **.001**	1.606(1.300-1.983)	< **.001**
Tumor size (>5 cm vs. ≤ 5 cm)	1.709(1.464-1.996)	< **.001**	1.510(1.261-1.808)	< **.001**	1.820(1.556-2.129)	< **.001**	1.537(1.288-1.835)	< **.001**
Encapsulation (present vs. absence)	0.767(0.605-0.973)	**.029**	/	/	0.907(0.722-1.141)	.406		
LNM (yes vs. no)	2.970(2.457-3.589)	< **.001**	2.274(1.838-2.813)	< **.001**	2.425(1.986-2.960)	< **.001**	1.969(1.580-2.454)	< **.001**
Macro-vascular invasion (yes vs. no)	1.440(1.053-1.968)	**.022**	/	/	1.841(1.346-2.519)	< **.001**	1.524(1.095-2.120)	**.013**
Micro-vascular invasion (yes vs. no)	1.921(1.532-2.409)	< **.001**	1.715(1.339-2.196)	< **.001**	1.621(1.293-2.032)	< **.001**	/	/
Differentiation (III-IV vs. I-II)	1.466(1.258-1.708)	< **.001**	1.521(1.278-1.810)	< **.001**	1.443(1.236-1.683)	< **.001**	1.401(1.184-1.658)	< **.001**

TB, total bilirubin; DB, direct bilirubin; γ-GGT, gamma-glutamyl transferase; CEA, carcinoembryonic antigen; CA19-9, carbohydrate antigen 19-9; LNM, lymph node metastasis
